# Women scientists in China: current status and aspirations

**DOI:** 10.1093/nsr/nwab101

**Published:** 2021-06-09

**Authors:** Chao Gu

**Affiliations:** Department of History of Science, Technology and Medicine, Peking University, China

## Abstract

Throughout history, gender inequality has persisted in most parts of the world. Since the founding of the People's Republic of China (PRC) in 1949, substantial progress has been made towards gender equality in China. Today, a large number of Chinese women scientists are making significant contributions to advance science. However, are they facing gender discrimination in hiring and promotion? Do they have access to the same opportunities as their male colleagues? What are the potential approaches to further promote gender equality in China's scientific community given myriad unfavorable social factors? Recently, *NSR* invited five Chinese female scientists and two gender experts to discuss these issues. Here are their observations and suggestions.

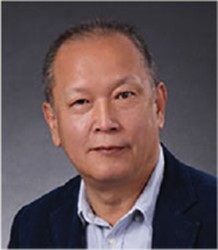

Bing Liu

Professor at the Department of the History of Science, Tsinghua University

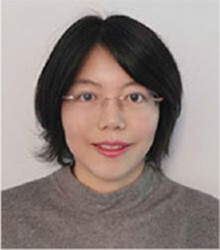

Jun Lu

Senior Engineer at Beijing Institute of Tracking and Telecommunications Technology, and Deputy Chief Designer of BeiDou Grounded Test and Validation System

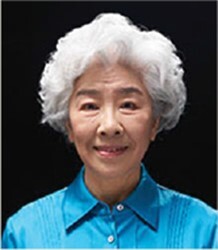

Chih-chen Wang

Professor at the Institute of Biophysics, Chinese Academy of Sciences

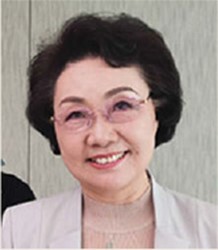

Hongyang Wang

President of the China Women's Association for Science and Technology (CWAST), Director of the National Center for Science in Liver Cancer

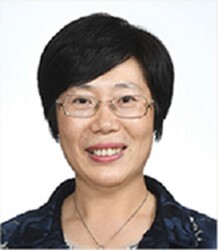

Xiaoyun Wang

C. N. Yang Professor at the Institute for Advanced Study, Tsinghua University

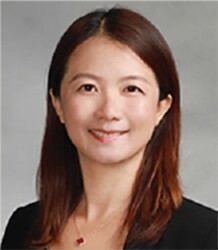

Yan Zheng

Chair Professor at the School of Environmental Science and Engineering, Southern University of Science and Technology

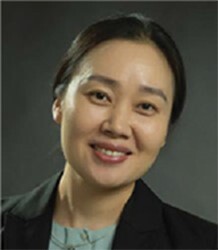

Wenpei Tang (Chair)

Professor at the School of Health Humanities, Peking University

## Gender issues in China's scientifIc community

**Wenpei Tang:** Professor C.C. Wang, as a senior female scientist, what's your opinion about gender and science in China? Is the scientific community in China gender-biased?

**Chih-chen Wang:** Everyone lives in his or her own time. I was born in 1942 and grew up in a completely different set of societal settings compared with today's generation. Thanks to PRC’s basic national policy of ‘gender equality’, I never suffered from gender discrimination as a young female scientist.

I was a little kid when PRC was founded in 1949, but I still have some impressions of women's social status and living conditions before that. I am deeply impressed by the great changes of China's society since 1949, including the impressive policies in promoting gender equality. Many deep-rooted gender-discriminative traditions were strictly banned, such as abandonment of baby girls, child brides, arranged marriage, mercenary marriage, and ghost marriage (to someone already dead). Moreover, nine-year compulsory education was applied to all kids, thus greatly boosting women's literacy. All of these indicate China's social progress. Actually, the social status of women can be a yardstick of the civilization of a country.

Since China began the ‘Reform and Opening Up’ period in the late 1970s, China's economy took off, and many people began to overzealously pursue personal wealth. The media also

went in a wrong direction that tempts some females to be vassals of the wealthy males. Thus, gender inequality rose again as a social trend in recent decades.

In most of the current TV series in China, female characters are stereotypically depicted as timid and lovable little women, like little birds resting upon men. I believe this is gender-biased and will have negative influences on the younger generation. When I was young, it was common sense that women can do whatever men can do. I never thought I couldn’t do anything because of my gender. But today, many people advocate that boys should be raised in a harsh way but girls should be raised tenderly. This will definitely harm the development and ambition of the girls. Also, many people say that girls are not good at STEM (science, technology, engineering and mathematics) and they should opt for ‘girl-specific’ jobs such as a secretary. Such social ethos is impacting the scientific community.

Gender inequality rose again as a social trend in the recent decades.

—Chih-chen Wang

**Hongyang Wang:** I agree that we should analyze China's situation from the angle of history. The PRC was established on the ruins of a semi-colonial semi-feudal society. In the old time, women had no position in both the society and the family. They didn’t have opportunities to realize their ambitions, and had no freedom in their marriage—in some areas, they were not allowed to dine at the table together with men. After the founding of PRC, dramatic changes have taken place. Currently, except for the few least-developed regions, such extreme gender discrimination practices have been eliminated and ‘women can hold up half the sky’. However, some gender issues still exist in China's scientific community, and the major problems are as follows.

First, there are a great number of women scientists, but many of them are working at lower-level positions, doing basic or supportive work. The space for their promotion is very limited, and very few were able to climb up to influential positions. According to data released in December 2019, women accounted for only ∼6% of the academicians of the Chinese Academy of Sciences (CAS) and ∼5% of the academicians of the Chinese Academy of Engineering (CAE). In total, the number of female academicians of CAS and CAE is less than 100 (out of∼1750). This ratio has been increasing over the years, but is still rather low. The underrepresentation of women in high-level roles is not restricted to CAS and CAE. The 2015 data released by the China Association for Science and Technology (CAST) revealed that women made up 21% of the members of all CAST academic societies. However, they made up only 13% of the society council members and 8% of the presidents and vice presidents.

I remember that once in an assessment meeting of a national high-level talent program, I was the only woman among the 30 reviewers and only one female candidate entered the last round of assessment. Some reviewers suggested that the female candidate should be eliminated. I felt obliged to oppose this unfair remark and obtained support from most experts. Since then, on many occasions, I have called for greater concern on the glass ceiling of women scientists. The National Natural Science Foundation of China (NSFC) became one of the first organizations that pay attention to gender equality issues, and now the proportion of females in its funding programs has risen considerably.

Second, women scientists have fewer opportunities compared with males with the same qualifications. By ‘opportunities’ here, I mean opportunities to get engaged in major research projects, to win honorary titles and so on. The competition for these projects or honors is often fierce, involving not only academic but also non-academic factors. When the non-academic factors are dominant, women are usually at disadvantage.

In terms of education and employment, women are also suffering from invisible gender discrimination. With all qualities equal, men have more opportunities than women. Facing female candidates, interviewers are inclined to think that they will not be able to concentrate on their work because of marriage, pregnancy and baby care. This is a result of social ethos in the division of labor in the family. It also implies that our public service system should be further improved to reduce the family burden of working women.

Third, in our scientific community, the leadership ability of women is often ignored. Fairness, integrity, preciseness, self-discipline, frugality and efficiency are highly valued leadership qualities, and women are often excelling in these qualities. But in reality, women have very limited chance to win leadership positions.

In many international scientific conferences, there must be at least one female keynote speaker, but in China this is not a common practice. Male-dominated conferences are everywhere. This will impede the career development of women scientists, and also damage the international image of China. We should initiate policies to provide equitable environment for women scientists, and to promote women to scientific leadership positions.

There are a great number of women scientists, but many of them are working at lower-level positions, doing basic or supportive work.

—Hongyang Wang

**Jun Lu:** Space technology and industry is often considered to be a ‘man's world’. But in China, women account for a rather high proportion of all space engineers, and many assume leading positions. Within the BeiDou Navigation Satellite System, there are three women sub-system chief designers, as well as a number of women deputy commanders and deputy chief-engineers. However, women are still rare among top leaders and academicians.

Space engineering is characterized by its high risk and long development cycle. So, I think women have some advantages in this field because of their qualities of being meticulous, dedicated, responsible and their ability to work under high pressure for a long time.

**XiaoyunWang:** Under Chinese laws and policies, women should enjoy equal rights with men. So, in my opinion, women should be able to overcome gender prejudices of the society and their families, and to pursue whatever kind of life they want. They should be able to pursue scientific research on the same footings as their male colleagues.

**Yan Zheng:** Gender inequality affects women not only in the scientific community, but also in industry, public service and many other professions. Women leaders are still rare. Although it's difficult to solve all gender issues of the entire society, steps can be taken within the scientific community to ensure equal opportunities for career advancement of all women researchers.

**Bing Liu:** Gender stereotypes exist worldwide, so we don’t need to shy away from this. In fact, admitting the problems, such as the glass ceiling of women scientists, is the first step towards improving the current situation.

I would like to note that in today's panel discussion, all of the women scientists are successful professors or even academicians, far exceeding the average level of the ordinary women scientists. As winners, you probably have not experienced much gender discrimination. But the grassroots women scientists may feel quite differently. They are the ones suffering from various gender barriers and lacking opportunities for positions, funding and promotion.

Success is a result of various inevitable and accidental factors. In a society, the population of the two genders is roughly the same, and in theory, men and women should have roughly the same likelihood of success. If the success rate of women is much lower, there must be something wrong. We need to figure out and address such problems.

In today's panel discussion, all of the women scientists are successful professors or even academicians, far exceeding the average level of the ordinary women scientists.

—Bing Liu

## Situations worldwide

**Wenpei Tang:** In terms of gender issues, what are the situations in other countries? Professor Yan Zheng worked abroad for many years, would you like to share some information?

**Yan Zheng:** Gender inequality also exists in scientific communities of other countries. Lower female representations of academicians, society fellows and plenary/keynote speakers in conferences can be seen on so many occasions. But the difference is that western countries have begun to recognize and address the gender issues, with actions taken much earlier than

Western countries have begun to recognize and address the gender issues, with actions taken much earlier than we had.

—Yan Zheng

we had. For example, the US National Science Foundation (NSF) launched the ADVANCE program in 2001. It has by now invested over $270 million to support women scientists and to encourage them to be heard. Additionally, universities and institutions also implemented changes, with some setting up on-site infant daycare facilities to ease childcare burdens. I am not as familiar with the European approach, on an awards committee that I served recently, it is shared that committees with decision-making authority should strive for no less than 40% of female representation, also known as the Gold Standard.

When I worked in Bangladesh for the United Nations Children's Fund on its poverty alleviation programs, I saw a completely different situation. On visits to rural villages, many adult women and girls would follow me around with great curiosity. In Bangladesh, there are many traditions and family rules restricting women's movement, behavior and interaction with men outside of their families. I could tell, just like us, they are eager to learn about the outside world. Unfortunately, except for the very few girls from wealthy families, most do not have opportunities to attend universities. Not surprisingly, there are also very few women scientists there.

I think female representation in the scientific community reflects how civilized a nation is. Clearly, there is still room for improvement to reach the Gold Standard in China! We need better policies. It is common that job advertisements still declare that only men are wanted. This should be prohibited by law.

**Hongyang Wang:** In Europe, gender equality is greatly emphasized. Women's equal opportunities for education and employment are basically guaranteed. Some countries even encourage parental leave of the fathers instead of the mothers. However, European women are also facing many challenges. I have studied and worked in Germany for 10 years. Many German female professors I know had no children. They are afraid that raising kids would interfere with their scientific career development.

**Chih-chen Wang:** But we can see many outstanding women politicians in Europe, such as Ursula von der Leyen, President of the European Commission. She is a successful politician as well as a mother of seven children. She organized the most gender-balanced executive team in the history of the European Union, with 13 women in this 27-commissioners team. Angela Merkel spoke highly of her excellent ability to reconcile work and family. I appreciate her very much!

**Bing Liu:** In humanities and social sciences, there are specific research disciplines focusing on gender issues. However, gender research, or Women's Study in China is relatively lagging behind, lacking enough researchers and social influence. Also, Women's Study has not been listed as an independent research discipline in China's universities. Regarding gender issues, we do need theoretical study based on China's specific societal context, without which, and with only personal feelings and appeals from the successful women professors, we may be able to provide opportunities for several young females, but it would not be possible to figure out fundamental solutions for the majority of women scientists.

## The underlying causes

**Xiaoyun Wang:** In my research group, I am willing to give more opportunities to my female students. But I found that fertility does greatly hinder their scientific research. Some would voluntarily give up research after childbirth, and it's rather difficult to re-start after two or three years’ gap. As for those who chose to continue, their time and energy are often much occupied by family issues, and it would be difficult for them to keep their focus sharp. Therefore, it's a big challenge for women to stay at the forefront of science after childbirth. Only the most outstanding talents are able to overcome the challenges.

We need to consider how to encourage and support them to balance work and life. I think all women scientists should believe in themselves that there is no insurmountable difficulty.

**Wenpei Tang:** Women are also trying to make some adjustments in their own way. For example, many would like to bear children when they are PhD candidates, to minimize the troubles they may encounter when looking for jobs in the future.

**Hongyang Wang:** Many smart women are not able to contribute much to the society because of the family affairs. We need to promote some solutions at the society level. For example, we can improve and perfect the services of kindergartens and nurseries, as well as the entire public service system.

Fertility does greatly hinder their scientific research.

—Xiaoyun Wang

**Bing Liu:** Gender issue is a social issue, touching upon cultural traditions and social mechanisms. For example, women usually shoulder more family responsibilities than men, so that their careers are more likely to be interrupted after childbirth. This is a result of the current family labor division. We should search for the social mechanisms that can provide a fair and favorable working environment for both genders.

Moreover, some of you mentioned that men and women have different personalities. Some of the feminine characteristics are advantages for scientific research, and some others, such as being sensible and emotional, may be disadvantages. But in fact, these personalities may not be ‘innate’ qualities, but are developed and disciplined by the society. Girls are often told to be tender, emotional and lovely, and should not be too calm, rational and independent. They are also repeatedly told about the ‘suitable majors and jobs for women’. Thus, when such a girl grows up and enters the scientific community, she may suddenly find that her shaped personalities are not suitable for scientific research.

Such problems cannot be solved by the scientific community itself. This needs the efforts of the entire society, including changes in policies and social ethos. Men and women should have equal access to social resources.

**Jun Lu:** I agree that the social and family labor division between men and women is different. In 2020, during the height of the COVID-19 pandemic, our institute implemented rigorous 24-hour access control. The researchers and engineers had to work and live in the office area and couldn’t go home for a month. If a husband and wife are both on our staff, one of them could stay at home to take care of their family while the other worked in the institute. It came out that almost all wives stayed at home when their husbands continued to work. On many occasions, men and women share the same social work responsibilities, while women shoulder much more family responsibilities at the same time—they are supposed to be the ones who take care of the elderly and kids.

**Yan Zheng:** That's right. Throughout the world, women tend to spend much more time on housework, but such labor is often unpaid and not recognized. Moreover, men and women are sometimes appraised differently for the same essential work. For example, my research involves extensive field investigations. In the eyes of many of my male colleagues both in China and in the US, I should be doing ‘real’ research instead of ‘playing’ out there. However, if it's a male researcher doing the same thing, he is very likely to be praised as an ‘explorer’.

**Wenpei Tang:** Another issue is women's freedom to choose their ways of life. Some argue that women have the freedom to give up their scientific career to pursue ‘more important things’ in their lives, such as maintaining a good family, nurturing their children or taking care of their parents.

But in fact, these ‘active choices’ may be forced or induced by the gender-biased education and social atmosphere. Girls are educated to be gentle, humble and tolerant, while boys are taught to be firm, brave and aggressive. Many women have internalized these mindsets. They become timid to venture into competitive work settings and ‘actively’ choose to come back to the family. But actually, this is a passive choice.

**Hongyang Wang:** When we say ‘women want equal opportunities’, we do not mean that every woman has to become top scientist. They can have their own choices. The point is that if they have the ability and willingness to do scientific research, they should be able to get equal opportunities to men.

Girls are educated to be gentle, humble and tolerant, while boys are taught to be firm, brave and aggressive. Many women have internalized these mindsets.

—Wenpei Tang

## The way forward

**Wenpei Tang:** We talked about the current situation and underlying causes of the gender issues in China's scientific community, and now I would like to invite you to provide some suggestions.

**Hongyang Wang:** First, we should keep appealing for gender equality. I do not appeal for myself—I have already got many opportunities, but for the many ordinary women facing unfair conditions. Gender inequality exists on many occasions and we should take the responsibility to cope with these issues.

Second, to cope with these challenges, we need to build a better policy system. There have been advances in China's scientific community. For example, the NSFC extended the age limit for female applicants: for Young Scientists Fund, the upper age limit is 35 for men and 40 for women; for Excellent Young Scientists Fund, it's 38 for men and 40 for women. The NSFC also stressed that women should be given priority when all other qualifications are equal. But there are still many other policies that can be adopted, for example, policies to relieve women's family burden, and to ensure a certain percentage of women speakers and women leaders.

**Chih-chen Wang:** Since NSFC’s extension of age limit, the male to female ratio of the Excellent Young Scientists Fund dropped to 5 : 1–4 : 1. While the ratio of the Distinguished Young Scholars Fund, with its age limit unadjusted, remained to be as high as 8:1. So, I believe that if we implement similar extensions to more funding programs, these programs will also become more gender-balanced.

Innovation is the basis of national power; talent is the basis of innovation, and women account for half of the talents. Therefore, we must further emphasize gender equality in our education system. This is the only way to help girls establish positive and scientific recognitions of the world and themselves, and grow up to be confident and independent women. The media orientation should also be changed to transfer the correct gender consciousness.

**Bing Liu:** Yes, gender education is very important, and we need theoretical research to guide gender education. A point is that gender education is not only for girls, but also for boys. Men are also victims of gender inequality. They are also disciplined by gender stereotypes and are suffering from severe pressure of social competition. Both genders need better understanding of gender equality.

Conceptually, gender education should not be simply telling the kids that men and women have no difference at all. It's probably better to acknowledge and give full play to the characteristics of both genders. Practically, we need to choose the proper strategies. In recent years, some radical feminists are very active in China. They call for the right concept of gender equality, but their activities are actually inciting gender antagonism in society, causing much trouble and making it harder to resolve the existing gender problems. This also caused the stigmatization of feminism.

The media should tell real and complete stories of women … they may encounter many obstacles and may also receive lots of support to overcome these obstacles.

—Jun Lu

**Xiaoyun Wang:** We should also encourage men to be more involved in their families, especially parenting. This would relieve pressure on women, and moreover, involvement from a father helps children tremendously.

**Wenpei Tang:** We studied the experience of a number of past-generation scientists in China, and found that almost all women scientists shared a commonality: they spent not much effort in family affairs. They are not married, or even if they married and had children, their families and husbands took up most of the family responsibilities. Without such strong support from the husbands, it would be difficult for them to be successful scientists.

**Bing Liu:** In the media, we often tell stories of these ‘lucky’ women scientists. But this may conceal the extra price that ordinary women often have to pay for their academic careers.

**Wenpei Tang:** Such propaganda set up role models that are unusual and not replicatable. If the media continues to tell the public that successful women scientists do not marry, have no children or are lucky enough to encounter a supportive husband, it would be a menace to the young women that are willing to be scientists.

**Jun Lu:** The media should tell real and complete stories of women: they have the ability to excel in various social positions; they need to strike a balance among different social and family roles; they may encounter many obstacles and may also receive lots of support to overcome these obstacles. We should not focus on the extremely lucky and untouchable sides of them.

**Wenpei Tang**: Thanks for your discussion. Scientists should join hands with scholars in humanities and social sciences, especially scholars of Science of Science, Sociology of Science and Gender Studies, to promote gender research in China and awaken more people's awareness of gender equality.

## Notes

Chao Gu is a postdoctoral researcher in the Department of History of Science, Technology and Medicine, Peking University, China.

